# Optimization of chemical texturing time for enhanced optical and electrical performance of Boron-Doped silicon solar wafers

**DOI:** 10.1038/s41598-025-27763-7

**Published:** 2025-11-29

**Authors:** Shimaa F. A. Amin, E. M.M. Ibrahim, Mohamed B. Zahran, Adham M. Nagiub

**Affiliations:** 1 Chemistry Department, Faculty of Science, Al-Azhar University in Assiut, Boy branch, Assiut, Egypt; 2 Physics department, Faculty of science, Sohag university, Sohag, 82524 Egypt; 3 Joint national Egyptian-Chinese Renewable energy laboratory, Sohag, Egypt; 4 PV Dep. Electronic institute Nozha elgedida, Cairo, Egypt

**Keywords:** Wet chemical texturing, P-type silicon, Solar cell, Optical, Electrical, Energy science and technology, Materials science, Optics and photonics, Physics

## Abstract

Surface texturing is a crucial step in enhancing the light-trapping efficiency of silicon solar cells by reducing optical reflection. The etching time plays a key role in determining the morphology and effectiveness of the textured surface. This study investigates the impact of chemical texturing duration on the structural, optical, and electrical properties of p-type boron-doped silicon wafers for solar cell applications. Texturing was performed using a mixture of potassium hydroxide and isopropyl alcohol (KOH-IPA) solution for varying durations (5–25 min). Structural, optical and electrical analysis revealed optimal pyramid formation at 20 min, coinciding with the lowest reflectivity and the lowest energy gap *E*_*g*_ (1.77 eV) and Urbach energy *E*_*u*_ indicating the most efficient absorption and reduced structural disorders. Also, conduction behavior transitions from DC-dominated at low frequencies to AC-dominated at higher frequencies, in agreement with the correlated barrier hopping (CBH) model. Maximum conductivity and dielectric loss were also observed in the 20-min etched sample and attributed to improved morphology and charge transport. These findings highlight 20 min as the optimal etching duration for enhancing photovoltaic efficiency by balancing light absorption and charge carrier dynamics.

## Introduction

Silicon solar cells dominate the photovoltaic sector due to their well-established technology and relatively high efficiency, reliability and cost-effectiveness; this makes them a leading choice for solar energy applications. As of 2021, they accounted for about 95% of photovoltaic production, with mono-crystalline silicon making up around 84% of that share^1^. Silicon is an indirect band gap semiconductor and has a high refractive index which is observed in its optical loss about 30–40%^2^. The maximum of valence band in silicon as indirect band gap presents in different *k* vector with the minimum of conduction band so, it needs to absorb a photon to change momentum and a phonon as an energy source which weakens its absorption. Many strategies are used to improve light absorption by silicon including lengthening the path length of light on the surface. Texturing as a precursor step of fabrication is a vital strategy for reducing losses and improving optical and electrical characteristics of silicon wafers through formation of pyramidal morphology by chemical etching^3^. Variable methods are used to etch silicon wafers physically and chemically. Chemical solutions may be isotropic acidic that is used for multi crystalline etching or anisotropic alkaline which is preferable for mono crystalline silicon wafer, the difference between them has been discussed in previously reported works^4–6^. The ability of wet chemical texturing in industrial application and high effective randomization makes it the most common way of texturing and the best way to enhance the light absorption^7^. Mixture of potassium hydroxide (KOH) and isopropyl alcohol (IPA) is an excellent solution for etching. KOH works as a main oxidant while IPA as a catalyst that has many functions where it enhances surface wettability, improves pyramid nucleation and also helps removing the resulted hydrogen bubbles from the reaction to avoid uniformity and coverage disrupt^8^. Although many trials have been addressed recently to replace KOH and IPA by other materials avoiding their problems, they still the conventional materials for use industrially^5,9^.

The pyramidal morphology of the surface achieves an efficient light randomization that makes Si surface as an “ideal Lambertian reflector”. Therefore, optimizing the texturing conditions is an excellent plan to enhance Si light trapping and endow higher photoelectric efficiency to the solar cell^3,10,11^. Texturing influence is not limited to tunning the optical properties; it has significant effect on the minority carrier life time enhancement and photoelectric conversion^12^. Accordingly, all photovoltaic parameters such as maximum voltage (*V*_*m*_,) maximum current (*I*_*m*_,) short circuit current (*I*_*sc*_), open circuit voltage (*V*_*oc*_) and fill factor (*Ff*) are affected and can be enhanced by texturing^13^. AC conduction in semiconductors is primarily influenced by defect states located within the mobility gap of these materials. Studying the AC conduction, offers valuable insights into the behavior of these defect states. In semiconductors, AC conductivity typically depends on frequency and most theoretical models that aim to explain AC conduction in disordered systems are based on the pair approximation. This approach attributes energy loss to electron transitions between defect states within the bandgap. The polarizability of an individual pair of defect states is analyzed, and the overall conductivity is obtained by summing the contributions from all such pairs^14^. Pollak and Geballe first applied the pair approximation to interpret AC conductivity in n-type crystalline silicon (c-Si)^15^. Later, Austin and Mott extended its application to amorphous materials, proposing that AC conduction arises from electron tunneling between localized states near the Fermi level a theory known as the quantum mechanical tunneling (QMT) model^16^. An alternative explanation is offered by the correlated barrier hopping (CBH) model, introduced by Elliott to describe AC conduction in chalcogenide glasses^17^, and later applied to silicon^18,19^. This model suggests that conduction occurs through hopping of electrons over potential barriers between defect states.

Investigation of the electrical conduction mechanism and dielectric properties in silicon as semiconductor and study the effect of texturing on optical and electrical parameters are essential tools to choose the optimum conditions. They are recently used in the electrical investigation of many types of solar cells like perovskite^20^ and silicon thin films^21^. Many factors moderate the resulted morphology of pyramidal texturing. Process time plays a vital role in etching rate and suitable reaction capacity monitoring. Short time achieve non completed process and also longer time possess over etching that affects negatively on cell electrical performance and production capacitance economically. Adama k.k. et al. investigated the effect of etching temperature and time as well as etchant concentration on the structural and optical properties of crystalline silicon wafers by the same used alkaline solutions. They also discussed the importance of optimum process time due to the formation of pyramidal grains with agglomerations of clusters which have their role of light trapping enhancement besides the effect of time on etching rate and surface roughness^9^. This work investigates the effect of texturing time on the structural, optical and electrical properties of p-type boron-doped silicon wafers. It aims to evaluate changes in key parameters such as reflectivity, optical bandgap, Urbach energy, dielectric constant and loss as well as the impedance and AC conductivity that reveal how light interacts with the surface and indicate the degree of disorder and the presence of localized states. The study gives approach to the conduction mechanism in chemically textured B-doped silicon and aims to investigate the effect of texturing time on the electrical properties of the wafers and the role of the formed defects.

## Experimental

### Texturing process

In the experimental procedure, commercial p-type boron-doped silicon wafers were cut using a manual scribing dicing in pieces with dimensions of 2 × 1 cm. Prior to cutting, the wafers were cleaned using solution of 10%KOH for 3 min at 70 °C, then they were cleaned by 2% hydrofluoric acid (HF) as hydrophobic step and lint-free cleanroom wipes were used to remove surface particulates. Following the cutting process, the samples were rinsed with deionized water and dried with a nitrogen stream. The resistivity of untextured wafers is between 0.6 and 1.8 Ω⋅m and its thickness is 180 μm.

Texturing was done by a mixture solution (KOH: IPA: H_2_O) (1gm: 7 ml:125 ml) for different times 5, 10, 15, 20 and 25 min at constant temperature 70^o^ C in ultrasound bath sonication device.

The reaction mechanism follows the equation^22^:

$$2{\text{KOH}} + {\text{Si}} + {{\text{H}}_2}{\text{O}} \to {{\text{K}}_2}{\text{Si}}{{\text{O}}_3} + 2{{\text{H}}_2}$$

where silicon is oxidized by KOH and produces soluble silicate with hydrogen gas. Finally, the textured wafers were again cleaned by hydrophobic process and then dried.

Reaction rate is estimated by weight loss percentage of the silicon wafers according to the following Eq^8^:

1 $${R_{{\text{av}}}} = \frac{{\Delta m}}{{2\rho st}}$$

where $\:\varDelta\:m$ is the weight loss, $\:\rho\:\:$is silicon density, *s* is surface area and *t* is the process time.

### Characterization

The surface morphology of the textured wafers was examined using field emission-scanning electron microscope (FE-SEM) model: ZEISS Sigma 500 VP FE-SEM. The optical properties were measured using UV spectrophotometer Model: JASCO 770 V. The impedance analysis, Ac conductivity and dielectrics were measured by LCR Meter model: HIOKI IM 3536.

## Results and discussion

**Fig. 1:**
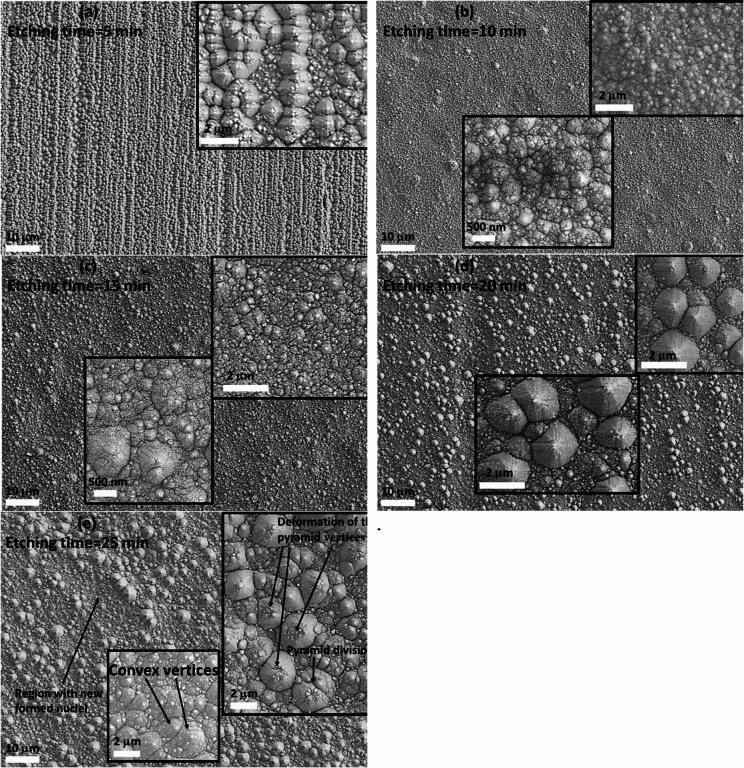
FE-SEM images of the wafers textured for (a) 5 min, (b) 10 min, (c) 15 min, (d) 20 min and (e) 25 min.

Figure 1 shows field emission-scanning electron microscope (FE-SEM) images for wafers textured by KOH and IPA mixture for 5, 10, 15, 20 and 25 min. Analyzing the changes in the surface morphology of the wafers enables to develop the scenario of the pyramidal shape formation by varying the etching time. Figure 1a indicates that by etching for 5 min, the surface of the B-doped Si wafer becomes rough. Deformed pyramids are observed on small parts of the surface but larger surface areas are covered by only small pyramid nuclei. Figure 1b shows that with texturing for 10 min, the wafer surface is fully covered by tiny pyramid nuclei and by elevating the reaction time to 15 min (Fig. 1c), pyramids start to grow taking their efficient pyramidal shape. After etching for 20 min, the surface is fully covered with randomly distributed pyramids with different size and clear sharp edges (Fig. 1d). The observed grown pyramids work as ideal Lambertian reflector and achieve an efficient absorption due to light randomization which matches well with the low reflectance observed for the wafer etched for 20 min as will be discussed latter. By elongating the etching time to 25 min (Fig. 1e), coverage of pyramids on the wafer surface is significantly improved, but significant change in the morphology of the pyramids is observed where the pyramids start to dissolute and divide into smaller collapsed octagonal entities with convex vertices. Destruction of pyramids is associated with starting new nucleation for growing new pyramids. Similar scenario has been reported by other research groups but with achieving complete surface texturing by etching using mixtures of KOH and IPA for 25–40 min depending on other conditions such as the etchant concentration and stirring method^23,24^. Chu et al.. reported that the coverage of pyramids on the wafer surface still incomplete until 15 min and growth of pyramids start after 15 min of etching process with significant enhancement in the pyramid spreading on the surface^25^. The study indicates also that with elevating the etching time, the pyramid growth and nucleation of new pyramids take place simultaneously and etching for time intervals longer than 20 min results in growing of new pyramids at the expenses of others with clear decrease in the uniformity and distribution of the pyramids on the surface. Previous studies also suggest that long etching process time has considerable effect on morphology of the pyramids where the vertices tend to form convex edges in coincidence with our results^26,27^.

**Table 1:** Values of weight loss, etching time, reflectance, energy gap, Urbach energy, slope of lnF(R) vs. hυ plots, exponent parameter and barrier height energy.

Texturing time	5 min	10 min	15 min	20 min	25 min
Weight loss	2.36%	4.6%	6.4%	8.5%	9.3%
Etching rate	0.0253	0.0246	0.0228	0.0225	0.01995
Reflectance	2.1–4.1%	0.65–1.78%	1.1–2.3%	0.6–1.7%	0.86–2.3%
Energy gap E_g_ (eV)	2.08	1.98	1.88	1.77	2.08
Slope of *lnF(R)* vs. hυ plots	1.428571	1.886792	2.380952	8.064516	4.347826
Urbach energy E_u_ (eV)	0.7	0.53	0.42	0.124	0.23
Exponent parameter (S)	0.88	0.89	0.38	0.5	0.7
Barrier height energy W_m_ (eV)	1.55	2.2	1.4	1.55	1.94

**Fig. 2:**
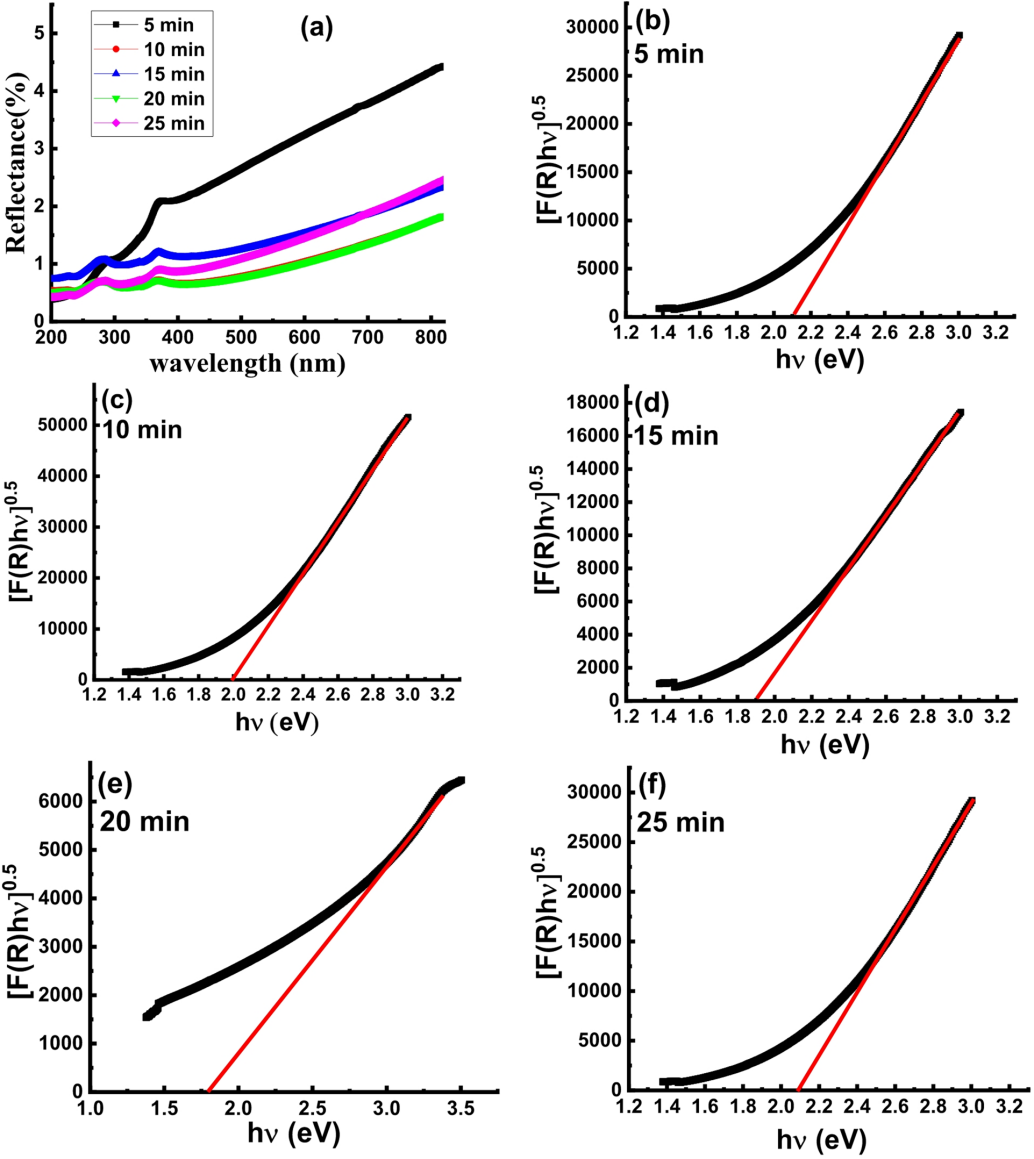
a) Reflectance% vs. wavelength spectra and b-f) [F(R)hυ]^1/2^ vs. hυ plots of the B-doped Si wafers textured for 5, 10, 15, 20 and 25 min respectively.

Reflectance% vs. wavelength spectra for the wafers textured for 5, 10, 15, 20 and 25 min are depicted in Fig. 2a. The wafer textured for 20 min exhibits the lowest values of reflectance over the whole range of the wavelength. This is in coincidence with the results of the FE-SEM investigations (Fig. 1d) where the 20 min textured wafer shows the most coverage surface of pyramids with lowest free-pyramids spaces among all other wafers. Besides, the grown pyramids have the best uniform morphology and distribution.

The aforementioned findings suggest that the reflectivity of the boron-doped Si wafers is significantly influenced by the time of the etching process. A key component of solar cells’ efficiency is their capacity to absorb a wide range of photons. In order to contribute to power conversion, photons are only absorbed when their energy surpasses the absorber layer’s band gap energy (*E*_*g*_). Thus, to moderate absorption and increase cell efficiency, the band gap must be optimized^28^. By using the data of the reflectance spectra, the Kubelka-Munk function F(R) is related to the absorption coefficient using the equation:

2 $$F(R) = \frac{{{{(1 - R)}^2}}}{{2R}} = \frac{{2a}}{S}$$

where *α* and *S* are the absorption coefficient and scattering coefficient, respectively.

Near the absorption edge, the relation between the optical energy gap (*E*_*g*_) and *F(R)* can be represented by Tauc’s equation:

3 $${[F(R)h\nu ]^{1/\gamma }} = {\beta _1}(h\nu - {E_g})$$

where *hυ* and *ß*_*1*_ are the photon energy and a constant, respectively while γ is a numerical parameter describes the mechanism of the electron transfer and equal to 0.5, 1.5 and 2 for allowed direct transition, forbidden direct transition and indirect transition, respectively. To determine the mechanism of the electron, transfer, the value of γ can be estimated from the following equation:

4 $$\:F\left(h\nu\:\right)=\frac{d\left(ln\:\alpha\:h\nu\:\right)}{d\left(h\nu\:\right)}=\frac{\gamma\:}{h\nu\:-{E}_{g}}$$

Equation 4 implies that the function *F(*hυ*)* reaches maximum value when the energy gap equal to the photon energy.

5 $$\:{\left[F\right(h\nu\:)}^{-1}]={\left[\frac{d\:\left(ln\:\alpha\:h\nu\:\right)}{d\:\left(h\nu\:\right)}\right]}^{-1}=\frac{h\nu\:}{\gamma\:}-\frac{{E}_{g}}{\gamma\:}$$

The value of γ was determined from the *[F(hν)]*^*−1*^ plots of the all textured wafers and found to be 2 indicating occurrence of indirect transition mechanism of the electron transfer as well known for the polycrystalline silicon^29^. The *[F(R)hυ]*^*1/2*^ vs. *hυ* plots in Fig. 2b-f exhibit linear behavior, indicating a good match to Tauc’s equation (Eq. 4). Table 1 lists the computed *E*_*g*_ values for the textured wafers. In general, it was discovered that the *E*_*g*_ values ranged from 1.77 to 2.08, which is comparable to those previously reported for boron-doped Si^30^. Nonetheless, it was shown that the *E*_*g*_ value is significantly influenced by varying the etching time. The highest *E*_*g*_ values are recorded for the wafer etched for 5 min due to the incompletely textured surface and that etched for 25 min due to the deformation of the pyramids surface as has been observed from the FE-SEM imaging. Noteworthy, these two wafers exhibit the highest reflectivity as seen in Fig. 2a. On the other side, the wafer that was etched for 20 min exhibits the lowest value of *E*_*g*_ = 1.77 eV, which is explained by the uniform and well-textured surface as shown from the FE-SEM images shown in Fig. 1d. The wafer etched for 10 min exhibits comparable reflectivity values to those of that etched for 20 min but with higher energy gap which may be attributed to the quantum confinement effect where it shows fully covered surface with pyramid nuclei in nanosized scale.

In solar cell systems, Urbach energy (*E*_*U*_) plays a crucial role in adjusting the voltage output and efficiency. *E*_*U*_ is correlated to impurity content and type, surface passivation, and structural disorders that link band tails with localized states. The observed change in the surface morphology throughout texturing process for various time intervals has effect on the Urbach energy value and may have an impact on the behavior of light absorption. The magnitude of *E*_*U*_ increases with the amount of defects present because increased defect content results in higher sub-bandgap energy levels into the forbidden energy gap. According to previously published simulation results^31^, the energy gap cannot be the only parameter that assess suitability for use a material in solar cell applications; but conjugation between *E*_*U*_ and *E*_*g*_ is a more practical way.

**Fig. 3:**
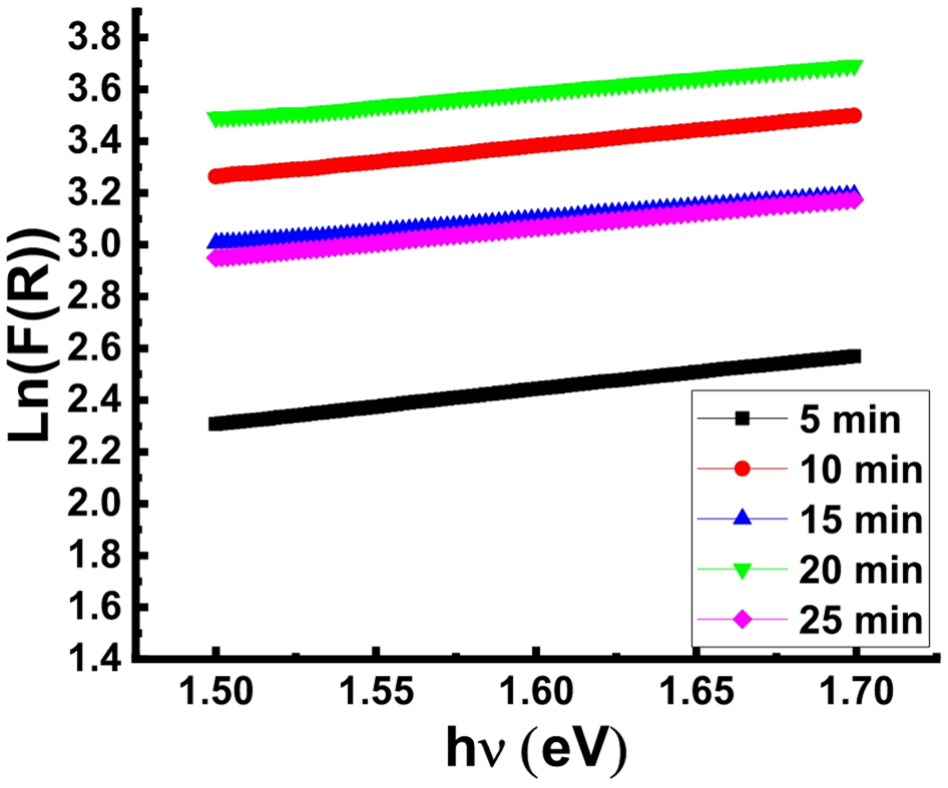
Ln(F(R)) vs. hυ plots of the B-doped Si wafers textured for 5, 10, 15, 20 and 25 min.

The relation between urbach energy and absorption coefficient is expressed by the following Eqs^31–33^. :

6 $$\alpha = {\alpha _0}\exp \left( {\frac{{h\nu }}{{{E_u}}}} \right)$$

7 $$F(R) = \frac{{{{(1 - R)}^2}}}{{2R}} = \frac{K}{S}$$

8 $$\ln F(R) = \ln {\beta _2} + \left( {\frac{{h\nu }}{{Eu}}} \right)$$

where ß_2_= $\:\frac{2{\upalpha\:}0}{S}$ = constant. *Eu* was calculated from *lnF(R)* vs. hυ plots (Fig. 3) and the values are tabulated in Table 1. The data imply that the *E*_*U*_ value is significantly influenced by the etching time and the wafer etched for 20 min shows the lowest value. The lowest *E*_*U*_ value of the 20 min etched wafer confirms the low deformation in the formed pyramidal morphology as has been suggested from the FE-SEM investigations.

**Fig. 4:**
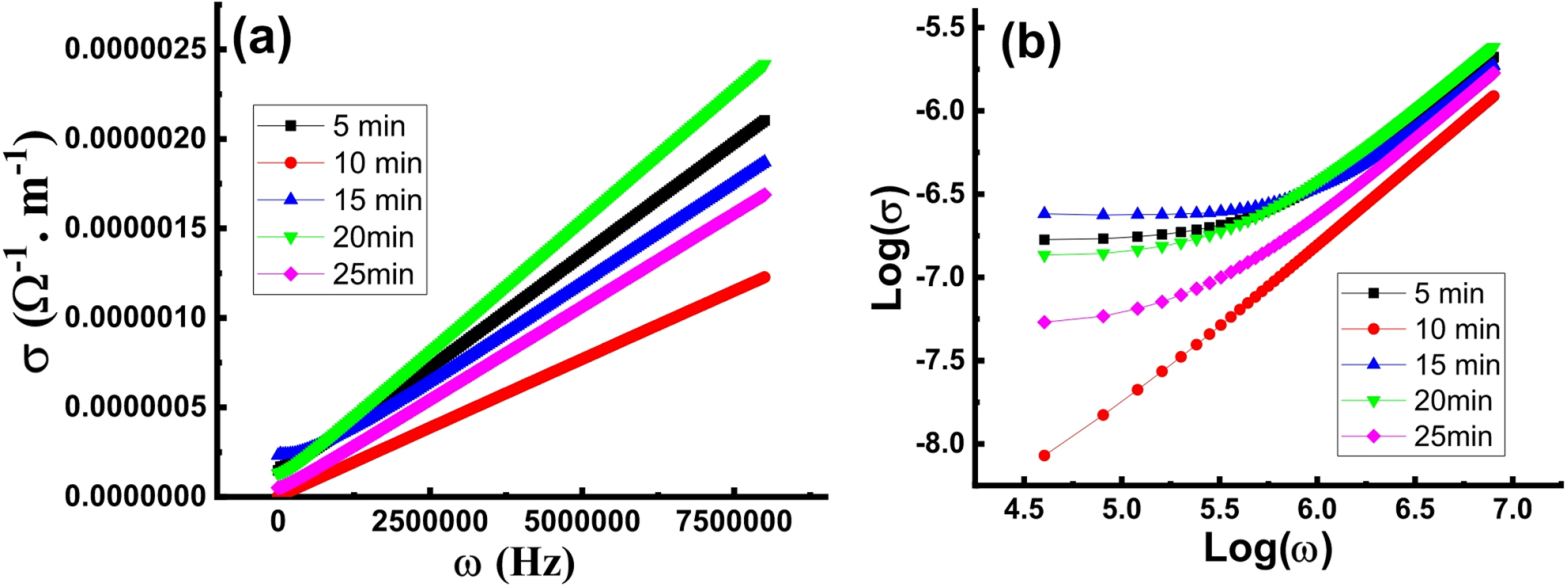
(a) σ vs. *ω* and (b) Log (σ) vs. Log (*ω*) plots of the B-doped Si wafers textured for 5, 10, 15, 20 and 25 min.

In order to study the effect of the etching time on the electrical properties and the mechanism that governs the electrical conduction, the frequency (ω) dependence of the electrical conductivity (σ) was measured for the B-doped Si wafers etched for 5, 10, 15, 20 and 25 min. The ω vs. σ plots (depicted in Fig. 4a) show that σ increases with ω in a typical feature of semiconductors. The total electrical conductivity is represented by the following equation as a sum of the DC conductivity (σ_DC_) and AC conductivity (σ_AC_)^34^:

9 $$\sigma = {\sigma _{{\text{DC}}}}(T) + {\sigma _{{\text{AC}}}}(\omega ,T)$$

*σ*_*AC*_ is a frequency dependent component that predominates in the high frequency range while *σ*_*DC*_ is a frequency independent component that predominates in the low frequency range. The data implies that the wafer etched for 10 min has the lowest conductivity which may be attributed to complete coverage of the wafer surface by pyramid nuclei with relatively smaller size and thus large numbers of inter-between boundaries that limits the charge carrier transfer across the surface. Noteworthy, the wafer that has been etched for 20 min shows the highest conductivity values within the higher frequency range of measurements. The *Log (σ)* vs. *Log (ω)* plots of the wafers etched for 5, 10, 15, 20 and 25 min depicted in (Fig. 4b) exhibit frequency-independent conductivity behavior over the low frequency range appears as a plateau region indicating predomination of the DC conduction mechanism. However, the conductivity increases sharply with increasing the frequency in the higher frequency range. This behavior indicates existence of two distinct conduction mechanisms, a frequency-independent mechanism based on band or band tail extended states conduction predominates in the low frequency range and a frequency-activated conduction mechanism by charge carriers hopping between defects which predominates at higher frequencies^35^.

In contrast, the wafer etched for 10 shows rapid increase in the conductivity upon increasing the frequency over the whole frequency range of measurements indicating domination of the AC conduction. The linear behavior of the *Log (σ)* vs. *Log (ω)* plots within the higher frequency range indicates that the σ_AC_ vs. ω dependency of the all wafers is well represented by Jonscher’s law^36^:

10 $${\sigma _{{\text{AC}}}} = A{\omega ^s}$$

where *s* is an exponent that denotes the type of conduction mechanism and A is a constant.

The electrical conduction mechanism can be explained in the framework of the correlated barrier hopping (CBH) model which was first presented by Pike where charge carriers hop between two defect centers that are separated by a potential barrier. Charge carrier hopping can take place either within a single defect potential well (intra-well hopping) or between two nearby defect potential wells (inter-well hopping)^37,38^. Chemical etching of the wafer surface always creates amorphous structures with numerous defects. Therefore, the observed variety of the measured electrical conductivity upon varying the etching time suggests the essential role of the etching time in controlling the electrical conduction in the wafers. Calculations illustrates that the exponent *s* has values lower than unity in coincidence with the CBH model (Table 1). Besides, the values of *s* of the samples under study are comparable to experimental and theoretical values reported by Grasso et al. for boron-doped silicon thin films^35^. The increase in the electrical conductivity upon increasing the frequency can be attributed to the increase of the hopping rate of the free charge carriers. The maximum barrier height over which the electron hop (*W*_*m*_) is related to the exponent *S* by the relation *W*_*m*_*=6k*_*B*_*T/(1-s)* where, *k*_*B*_ is the Boltzmann constant, *T* is the absolute temperature. As seen in Table 1, *W*_*m*_ values are close to the values of the optical energy gap. According to the model proposed by Elliot^19^, the barrier height energy *W*_*m*_ depends on the energy position between two charged centers that could lie almost symmetrically near the band edges. Therefore, the close values of *W*_*m*_ to the energy gap can be envisaged. The wafers etched for 10 and 20 min show the highest and lowest *W*_*m*_ values which is the reason of the lowest and highest conductivity values, respectively.

**Fig. 5:**
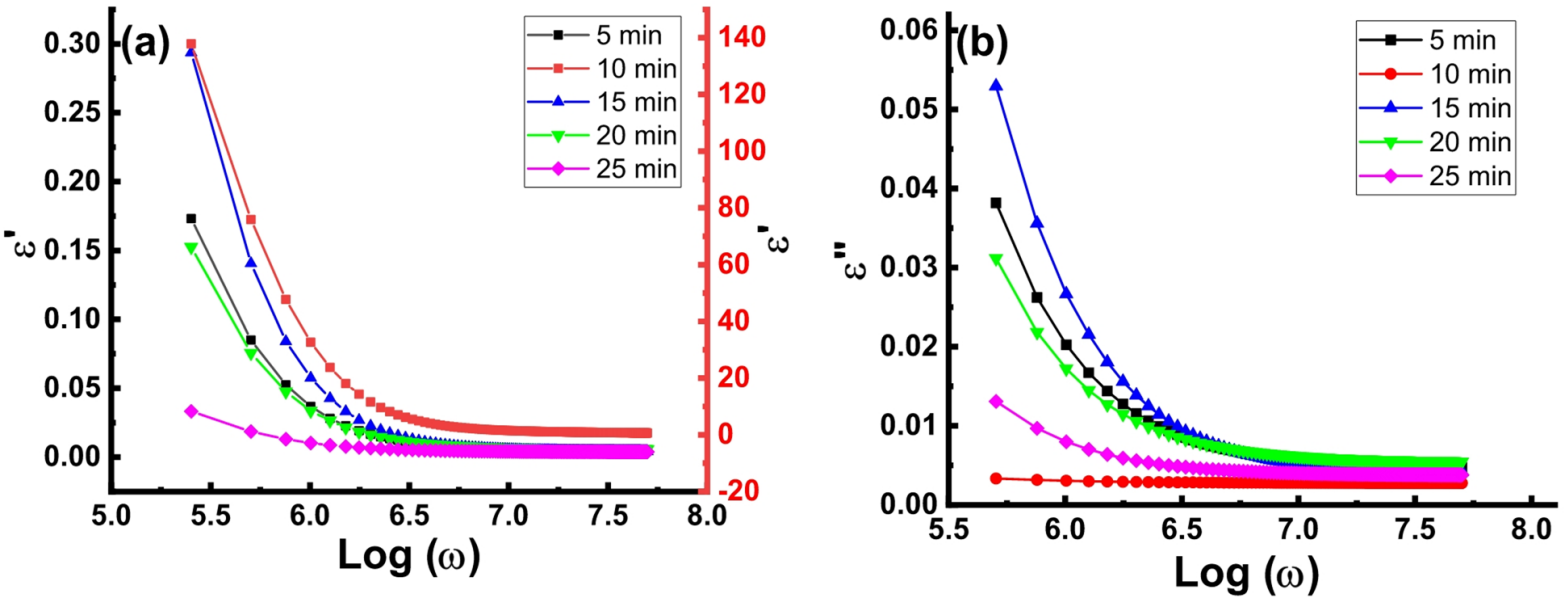
a) *ε*’ vs. Log (*ω*) and b) *ε*’’ vs. Log (*ω*) plots of the B-doped Si wafers textured for 5, 10, 15, 20 and 25 min.

The real permittivity (*ε’*) versus frequency plots are depicted in Fig. 5a. Generally, the frequency dependency of *ε’* show similar behavior for all the wafers where *ε’* dramatically decreases upon increasing *ω* in the low frequency range but showing approximately constant value at high *ω*. Various polarization mechanism including electronic, ionic, space charge and orientational polarization can contribute to the overall polarizability of a material^39,40^. Ionic and electronic polarizations are excluded because they remain up to and above the infrared frequency range. Only the space charge polarization due the travelling charge carriers and the orientational polarization due to the dipoles orientation can contribute to the overall polarizability.

The space charge polarization is caused by the defects where diploe moments are formed due to trapping the charge carriers at the defects. At low frequency, the dipole moments orient themselves easily in the direction of the external electric field leading to high values of the dielectric constant. At high frequencies, *ε’* approaches a constant value and all plots merge irrespective of the etching time. According to the Maxwell–Wagner polarization model based on Koops’ theory, the polarization process takes part due to charge movement into the material upon applying external electric field^41,42^. Koops’ theory states that the solid material is composed of highly conducting grains with low dielectric constant, separated by grain boundaries that are poorly conductive. The variation in the conductivity between these regions influences the dielectric behavior of the material. When charge carriers travel through a conductive region and encounter a grain boundary, their movement is disrupted, causing them to accumulate at the interface and forming space charge polarization^43,44^. As a result, the grains and grain boundaries function like a capacitor due to the formation of a Schottky barrier. When an electric field is applied, molecules rotate to align with the field, producing a net dipole moment per molecule and leading to a high dielectric constant (*ε*’). This mechanism also accounts for the observed high *ε’* for the wafer etched for 10 min, as increased grain boundary content that linked to the nanosized pyramid nuclei cover the surface (seen in FE-SEM investigation) enhances polarization^45^. However, at higher frequencies, the dipoles can no longer respond to the rapidly changing field, causing a reduction in orientation polarization. Consequently, *ε’* decreases to a steady value, where only the frequency-independent space charge polarization contributes to the overall polarization^46^.

Figure 5b illustrates how the imaginary part of the dielectric constant (*ε*’’) varies with frequency for the boron-doped silicon wafers under study. In general, *ε’’* decreases with increasing frequency and eventually reaches constant value at higher frequencies. Dielectric loss, which represents energy dissipated as heat when an AC electric field is applied, mainly occurs due to the delayed response of the material to the external field. At low frequencies, the movement of charge carriers is more significant, contributing heavily to dielectric loss and resulting in high *ε’’* values. As seen from Fig. 5b, the wafers etched for 15 min shows the highest dielectric loss which can be attributed to relatively easy movement of the electric charge due to the lowest defects content. As frequency increases, the impact of charge carrier’s migration diminishes, leading to a drop in *ε’’*. Noteworthy, the wafers etched for 10 min shows the lowest dielectric loss indicating limited carriers migration in well coincidence with the behavior of the electrical conductivity depicted in Fig. 4. The 20 min etched wafer shows the highest dielectric loss within the higher frequency range due to the large mean free path of the charge carriers because the large size of pyramids that cover the surface (as seen from FE-SEM investigations) permits charge carriers movements in larger distance before accumulation on the grain boundaries.

**Fig. 6:**
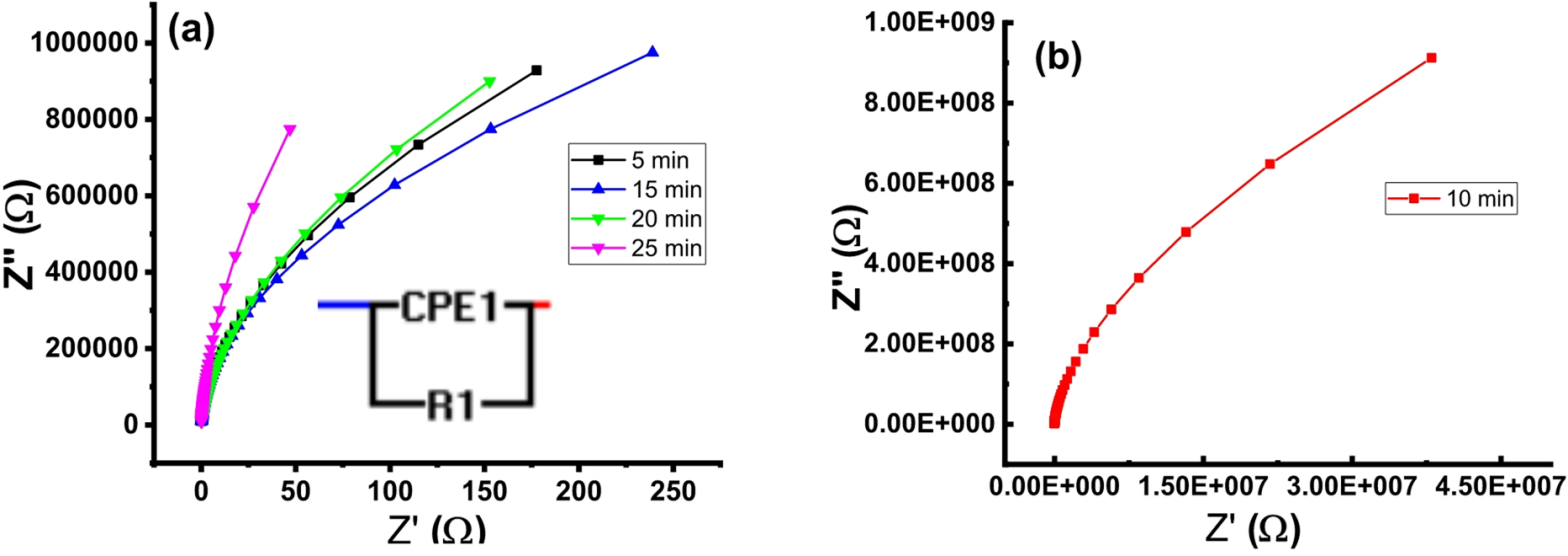
(a) The Cole–Cole plots of B-doped Si wafers etched for 5, 15, 20 and 25 min (inset is the equivalent circuit) and (b) 10 min.

Complex impedance spectroscopy plays a vital role in examining the behavior of mobile and bound charges within a material. It helps distinguish the contributions of grains, grain boundaries, and interfaces to the overall polarization mechanism. As previously discussed, grain effects are more dominant at higher frequencies, whereas grain boundary effects become more significant at lower frequencies. The total impedance of a material can be represented as^34^:

11 $$Z = \frac{R}{{1 + jRCW}} = Z' - jZ''$$

12 $$Z' = \frac{R}{{1 + {{(CRW)}^2}}}for{\text{ }}real{\text{ }}part\;\;\;$$

13 $$Z'' = \frac{{RCW}}{{1 + {{(RCW)}^2}}}for{\text{ }}imaginary{\text{ }}part$$

where *Z’* denotes the real part of impedance, corresponding to resistance, and *Z’’* represents the imaginary part, which relates to capacitance. Figure 6 displays Cole–Cole (Nyquist) plots for the B-doped Si wafers. These plots show a dispersed nature rather than perfect semicircles centered on the real axis, suggesting a non-Debye relaxation behavior. A noticeable variation in the semicircle radii with texturing for different time intervals is observed indicating a change in the resistance with varying the etching time due to the accompanied alteration in the ability of the charge carriers transfer. The inset of Fig. 6a illustrates the equivalent circuit comprising in-parallel bulk resistance (R_b_) and a constant phase element (CPE) that describes the microstructural–electrical relationship. The impedance of the CPE is expressed as^47^:

14 $${Z_{{\text{CPE}}}} = \frac{1}{{Q{{(i\omega )}^n}}}$$

where *Q* and *n* denote the magnitude of the admittance at 1 rad/s and the phase factor, respectively. The CPE behaves as an ideal capacitor at *n* = 1 and as a pure resistor at *n* = 0. From the fitting parameters (Table 2), the 20-min etched wafer shows the lowest R_b_ value suggesting the formation of more interconnected conductive pathways due to the well coverage of pyramids on the wafer surface^47^. The proposed equivalent circuit demonstrates good agreement with the experimental impedance data.

**Table 2:** The equivalent circuit parameters.

Texturing time	*R*_b_	Q	*N*
5 MIN	1.0099 × 10^6^	1.9984 × 10^− 11^	0.79995
10 MIN	1.01 × 10^6^	5.7795 × 10^− 11^	2.9301
15 MIN	1.01 × 10^6^	4.5228 × 10^− 11^	0.12169
20 MIN	1.0042 × 10^6^	1.9873 × 10^− 11^	0.79961
25 MIN	1.0098 × 10^6^	1.9941 × 10^− 11^	0.79984

## Conclusion

This study establishes that the duration of chemical texturing using a KOH–IPA solution is a decisive factor governing the structural, optical, and electrical performance of boron-doped silicon wafers. The results collectively demonstrate that a 20-minute etching time produces an optimally textured surface characterized by uniformly distributed pyramidal structures, the lowest reflectivity, and the smallest energy bandgap and Urbach energy indicating enhanced light absorption and reduced structural disorder. Electrical and dielectric analyses further confirm that charge transport in the textured wafers follows the correlated barrier hopping (CBH) model. The 20-minute etched sample exhibits the highest AC conductivity and dielectric loss at elevated frequencies due to improved carrier mobility and well-formed conductive pathways. These interrelated structural, optical, and electrical enhancements highlight that precise control of etching duration is crucial for achieving maximum photovoltaic efficiency. From an industrial perspective, the identification of 20 min as the optimal texturing time provides a practical and cost-effective guideline for large-scale silicon wafer processing, enabling improved light management without compromising throughput. Future studies could focus on scaling this process to larger wafer formats, exploring environmentally benign alternatives to IPA, and integrating this optimized texturing step with advanced passivation or anti-reflective coatings to further enhance solar cell efficiency and durability.

## Data Availability

the authors declare that the data supporting the findings of this study are available within the paper.
